# Diabetic Retinopathy Fundus Image Classification and Lesions Localization System Using Deep Learning

**DOI:** 10.3390/s21113704

**Published:** 2021-05-26

**Authors:** Wejdan L. Alyoubi, Maysoon F. Abulkhair, Wafaa M. Shalash

**Affiliations:** 1 Information Technology Department, King Abdulaziz University, Jeddah 21589, Saudi Arabia; mabualkhair@kau.edu.sa (M.F.A.); wshalash@kau.edu.sa (W.M.S.); 2 Computer Science Department, Faculty of Computers and Artificial Intelligence, Benha University, Benha 13518, Egypt

**Keywords:** computer-aided diagnosis, convolutional neural networks, deep learning, diabetic retinopathy, diabetic retinopathy classification, diabetic retinopathy lesions localization, YOLO

## Abstract

Diabetic retinopathy (DR) is a disease resulting from diabetes complications, causing non-reversible damage to retina blood vessels. DR is a leading cause of blindness if not detected early. The currently available DR treatments are limited to stopping or delaying the deterioration of sight, highlighting the importance of regular scanning using high-efficiency computer-based systems to diagnose cases early. The current work presented fully automatic diagnosis systems that exceed manual techniques to avoid misdiagnosis, reducing time, effort and cost. The proposed system classifies DR images into five stages—no-DR, mild, moderate, severe and proliferative DR—as well as localizing the affected lesions on retain surface. The system comprises two deep learning-based models. The first model (CNN512) used the whole image as an input to the CNN model to classify it into one of the five DR stages. It achieved an accuracy of 88.6% and 84.1% on the DDR and the APTOS Kaggle 2019 public datasets, respectively, compared to the state-of-the-art results. Simultaneously, the second model used an adopted YOLOv3 model to detect and localize the DR lesions, achieving a 0.216 mAP in lesion localization on the DDR dataset, which improves the current state-of-the-art results. Finally, both of the proposed structures, CNN512 and YOLOv3, were fused to classify DR images and localize DR lesions, obtaining an accuracy of 89% with 89% sensitivity, 97.3 specificity and that exceeds the current state-of-the-art results.

## 1. Introduction

Diabetic retinopathy (DR) is a common diabetes complication that occurs when the retina’s blood vessels are damaged due to high blood sugar levels, resulting in swelling and leaking of the vessels [1]. In an advanced DR stage, the vision may be lost completely. The percentage of blindness worldwide resulting from DR is 2.6% [2]. Therefore, diabetes patients need regular screening of the retina to detect DR early, manage its progression and avoid the risk of blindness.

**Figure 1:**
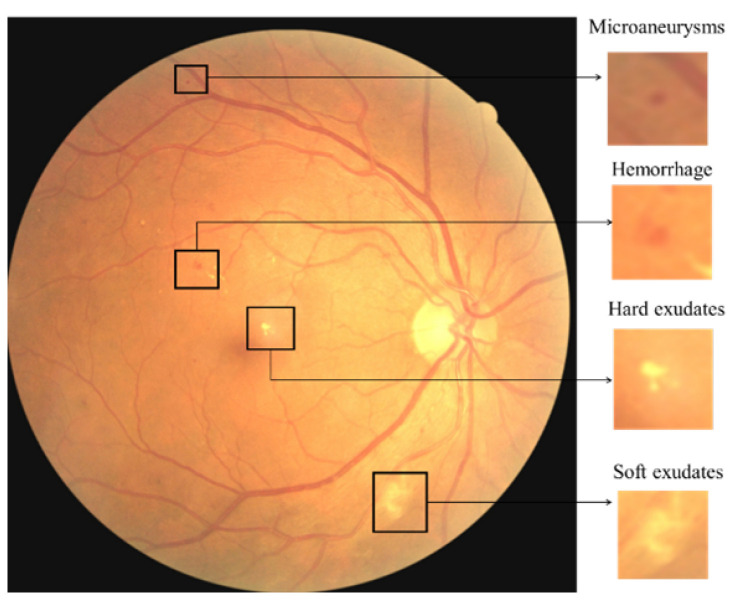
The different types of DR lesions.

**Figure 2:**
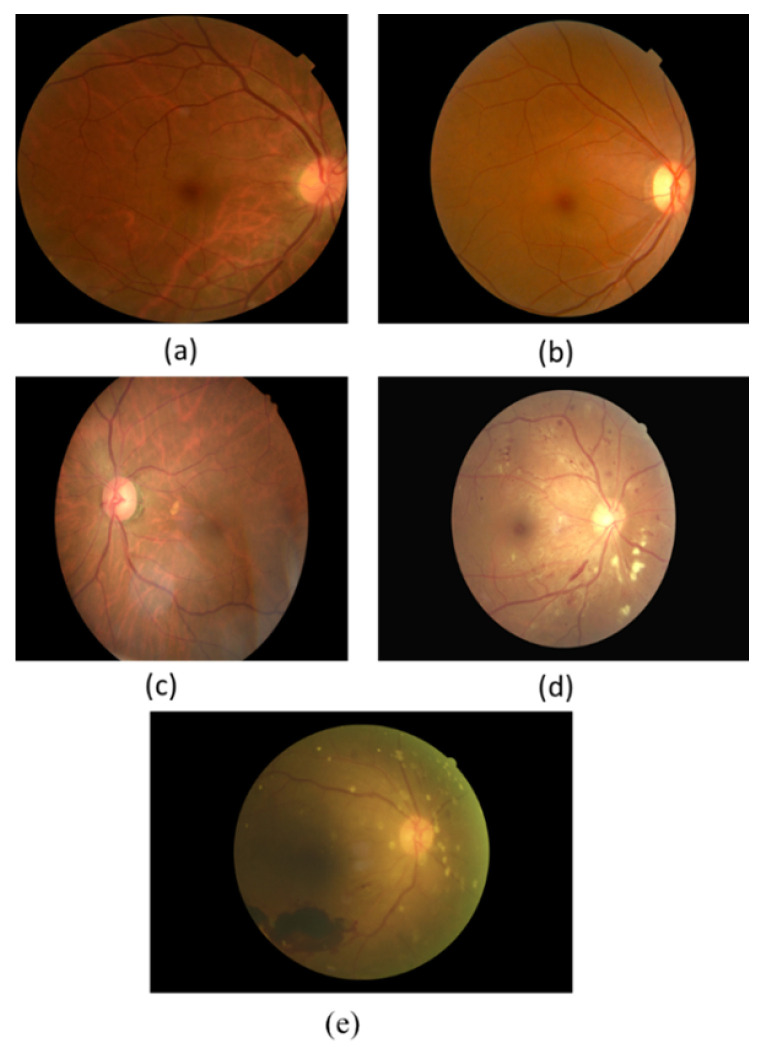
The DR stages: (**a**) No DR (**b**) Mild, (**c**) Moderate, (**d**) Severe, (**e**) Proliferative DR.

The leaking blood and fluids appear as spots, called lesions, in the fundus retina image. Lesions can be recognised as either red lesions or bright lesions. Red lesions involve microaneurysms (MA) and haemorrhage (HM), while bright lesions involve soft and hard exudates (EX) as shown in Figure 1. The small dark red dots are called MA and the larger spots are called HM. Hard EX appears as bright yellow spots, while soft EX, also called cotton wool, appears as yellowish-white and fluffy spots caused by nerve fiber damage [3]. The five DR stages depend on the types and numbers of lesions on the retina image, as shown in Table 1. Samples of the various DR stages (no DR, mild DR, moderate DR, severe DR, and proliferative DR) are shown in Figure 2.

The manual diagnosis of DR by ophthalmologists is time-consuming, requires considerable effort, and is prone to disease misdiagnosis. Therefore, using a computer-aided diagnosis system can avoid misdiagnosis and reduce overall cost, time and effort. During the last decade, deep learning (DL) approach has emerged and been adopted in many fields, including medical image analysis. DL can identify features accurately from input data for classification or segmentation and typically outperforms all traditional image analysis techniques. DL techniques does not need to extract the hand-crafted features while it requires extensive data for training [5]. In contrast, machine learning techniques require extraction of the hand-crafted features, but they do not need extensive data for training. In DR detection, the machine learning techniques need to extract the vessel firstly, as in [6,7]. Then, extract DR lesions’ features for classification as in [8]. DL applications include the segmentation, classification, retrieval, detection and registration of the images [9]. Convolutional Neural Network (CNN) is a type of DL method that is a widely used [9], highly effective and successful method for image analysis [10,11].

There has been a considerable number of efforts to automate DR image classification using DL to help ophthalmologists detect the disease in its early stages. However, most of these efforts focused only on detecting DR instead of detecting various DR stages. Moreover, there have been limited efforts to classify and localize all the DR lesions types, which is very helpful in practice, as ophthalmologists can evaluate DR severity and monitor its progression based on the appearance of these lesions. For these reasons, we propose a fully automated screening system using CNN to detect the DR five stages and localize all DR lesion types simultaneously. The proposed system helps ophthalmologists mimic their DR diagnosis method, which localizes DR lesions, identifying its type and determining the DR exact stage. The current study investigates three CNN-based models to classify the DR images into stages. The first model was designed using transfer learning by fine-tuning EfficientNetB0 [12]. The other two models, CNN512 and CNN229, were designed, tuned and trained from scratch. For DR lesions localization and classification, a tuned YOLOv3 [13] model was used. To achieve the best DR stages classification result, the image classification model and the DR lesions localization model were fused. We investigate many CNN structures to classify and localize DR images’ lesions until it reaches the best combination of a CNN and YOLOv3 structure to present a fully automatic DR grading and localization system. The present study’s main contribution is the promising new design and fusion of two models to construct the proposed screening system. The first structure is the CNN512, a CNN designed, tuned and trained from scratch to classify each image according to one of the DR stages. While the second is a modified YOLOv3 to localize its DR lesions simultaneously. The proposed system shows a promising result.

As far as we know, YOLOv3 has been used in the detection of the red lesion as in [14]. The novelty of the current work is considered the first research used YOLOv3 to detect the different DR lesions.

This paper is structured as follows: Section 2 briefly analyses deep learning based related works on DR stages and lesions detecting, while Section 3 presents the materials and proposed methods. Section 4 describes the experiments and results. The discussion and conclusion are presented in Section 5 and Section 6, respectively.

## 2. Related Works

CNN has been used widely in the classification and localisation of retinal fundus images. The DR detection works using DL can be categorized into three main categories: binary DR classification, multi-level DR classification and hybrid classification. In the following sections, we will summarise the recent efforts in DR classification in these three categories. A comparison between the related works is presented in Table 2.

### 2.1. Binary Classification

This section looks at the studies that have classified DR images into two categories. Pires et al. [15] proposed a custom CNN to detect referable DR images and non-referable DR images. Their CNN were trained on the Kaggle [16] and achieved an AUC of 98.2% on the Messidor-2 [17]. Jiang et al. [18] created a new dataset to classify DR images to referable DR or not using three pretrained CNNs; Inception-Resnet-V2 [19], Inception V3 [20] and Resnet152 [21]. These CNNs were combined using the Adaboost algorithm. They obtained an AUC of 0.946. Liu et al. [22] created a weighted paths CNN called WP-CNN to classify referable DR images in a private dataset. They reported an accuracy (ACC) of 94.23%. Das et al. [23] proposed two independent CNN to classify the images into normal or DR images. Their CNNs obtained an ACC of 98.7% on the DIARETDB1 dataset. Although the previous studies achieved good results in detecting DR, they did not take the five DR stages and the various lesions into account. The main drawback of the binary classification method is that it only classifies the DR images into two categories, without considering the five DR stages. The identification of the exact DR stages is essential in selecting a suitable treatment process and preventing retina deterioration.

### 2.2. Multi-Level Classification

This section reviews the works that have classified DR images into various stages. Wang et al. [24] examined the performance of three pre-trained CNNs in the Kaggle dataset [16] to classify all the stages of the DR images. The three CNN architectures used were InceptionNet V3 [20], AlexNet [39] and VGG16 [40]. The best average ACC of 63.23% was obtained by InceptionNet V3. The work of [25] transferred learning pre-trained AlexNet [39], VggNet [40], GoogleNet [41] and ResNet [21] to detect the different DR stages in the Kaggle dataset [16]. Their results showed that VggNet achieved the higher ACC, with a value of 95.68%. Mobeen-ur-Rehman et al. [26] proposed a simple CNN to detect the DR stages of the Messidor dataset [17]. Their CNN obtained an excellent ACC of 98.15%. Zhang et al. [27] proposed a method to detect the DR stages of their private dataset. They fine-tuned InceptionV3 [20], ResNet50 [42], Xception [43], InceptionResNetV2 [19], and DenseNets [44] and then combined the strongest CNNs. This method obtained an ACC of 96.5%. Harangi et al. [28] classified the DR stages by integrating hand-crafted features and AlexNet [39]. They used the Kaggle dataset [16] for training and the IDRiD [45] dataset for testing. This method achieved an ACC of 90.07%. Shanthi and Sabeenian [29] used Alexnet [39] to classify the DR stages of the Messidor dataset [17]. Their ACC was 96.35%. Li et al. [30] used ResNet50 [21] with attention modules to classify the stages in the IDRiD dataset [45], resulting in a 65.1% joint ACC. Dekhil et al. [31] transferred learning VGG16 [40] to classify the DR stages in the APTOS 2019 Kaggle dataset [46], and they achieved an ACC of 77%. He et al. [32] proposed a CABNet network to classify DR images into stages, achieving an ACC of 85.69% in the DDR [37]. Kassani et al. [33] modified Xception model [43] to classify the DR stages in the APTOS 2019 Kaggle dataset [46], resulting in a 83.09% ACC. Bodapati et al. [34] proposed a composite network with gated attention to classify DR images into stages, achieving an ACC of 82.54% in the APTOS 2019 Kaggle dataset [46]. Hsieh et al. [35] trained the modified Inception-v4 [19] and the modified ResNet [21] to detect any DR, proliferative DR and referable DR in their private dataset and the EYEPACS dataset. They obtained an AUC of 0.955, 0.984 and 0.955, respectively in detecting any DR, proliferative DR and referable DR.

**Figure 3:**
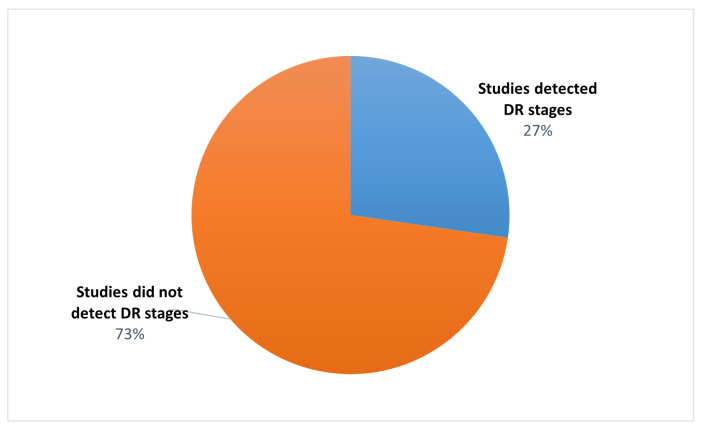
The ratio of studies that classified the DR stages [47].

These previous studies demonstrated that CNN is effective in classifying DR images. However, localising DR lesions with DR image classification is more efficient for ophthalmologists at diagnosis. Moreover, Alyoubi et al. [47] reported that most of the studies, almost 70%, classified the fundus images using binary classifiers such as DR or non-DR, while only 27% classified the input to one or more stages, as shown in Figure 3.

### 2.3. Hybrid Classification

This section presents the studies that classified DR images and localised lesions at the same time. Zago et al. [36] used VGG16 [40] to detect red lesion patches of the DR images, and then they classified the image to DR or no-DR based on the detected red lesions. Their best results were achieved in the Messidor dataset [17] with an AUC of 0.912. Li et al. [37] created a dataset called the DDR to classify images into five DR stages and to localise lesions. For the stages classification, they achieved the best ACC of 82.84% using SE-BN-Inception [48], while for localisation, they achieved a mAP of 9.2 using Faster RCNN [49]. Wang et al. [38] used two modified RFCN [50] to detect the stages of DR and localise the MA and HM. Then the results from the two RFCN were merged. In their private dataset, this method achieved a mAP of 92.15 in localizing, while in classification, they achieved a 92.95% ACC.

**Figure 4:**
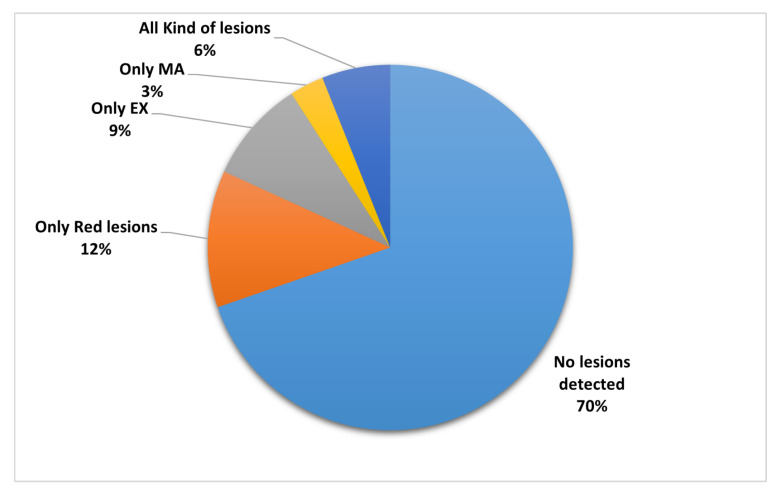
The ratio of studies that classified the DR lesions [47].

Many studies, such as those by W. Alyoubi et al. [47] and T. Li et al. [51], show that the main limitation of the DR classification systems is that only a limited number of the studies detected and localized the types of DR lesions on the fundus image, as shown in Figure 4. Furthermore, there are limited studies that detected the DR stages, grading and lesions together. Lesions localization with high accuracy helps with grading the cases and the patients’ follow-up, which is considered a critical requirement for DR patients.

## 3. Materials and Methods

**Figure 5:**
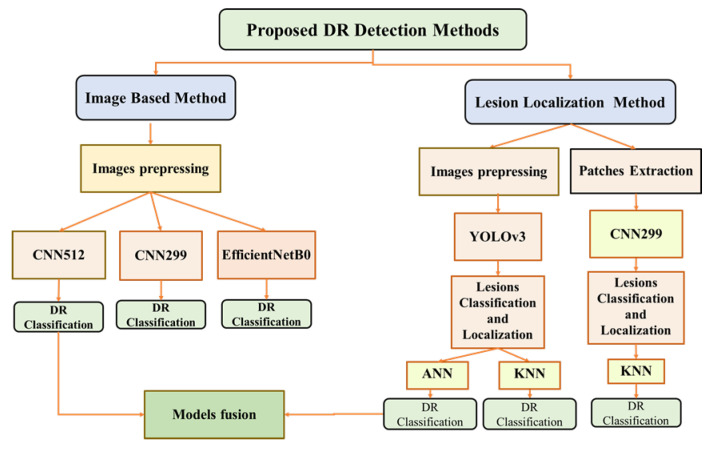
Block diagram of the different proposed models for DR images classification and localization.

This section presents the datasets and the preprocessing methods used in the current work. Moreover, it explains the two proposed methods, shown in Figure 5, to classify the DR stages and localise the DR lesions types. The first method, called the Image-Based Method, utilises the whole preprocessed RGB retina images as an input for the CNN, while the second method, called Lesion localization method, is based on the lesions detection in order to classify the images into the five DR stages.

### 3.1. Datasets

Two publicly available fundus retina datasets were used in this work: the DDR [37] and Asia Pacific Tele-Ophthalmology Society (APTOS) 2019 Kaggle [46]. Table 3 shows more details about these datasets.

- The DDR dataset [37] consists of 13673 fundus images acquired at a 45${}^{\circ }$ field of view (FOV). Among these, there are 1151 ungradable images, 6266 normal images, and 6256 DR images. There are 757 images annotated by providing a bounding box for lesions (MA, HM, hard EX, and soft EX) to locate all DR lesion types. The dataset has different image sizes, classified to five DR stages and split into train, valid, and test images. The distribution of the dataset is imbalanced in that the normal images are more than the DR images. The annotated lesions distribution is shown in Table 4.
- The APTOS 2019 Kaggle dataset [46] consists of 3662 retina images with different image sizes. Only the ground truths of the training images are publicly available. The dataset is classified into five DR stages. In addition, 1805 of the images are normal and 1857 are DR images. The distribution of the dataset is imbalanced, with most of the images normal.

### 3.2. Preprocessing

**Figure 6:**
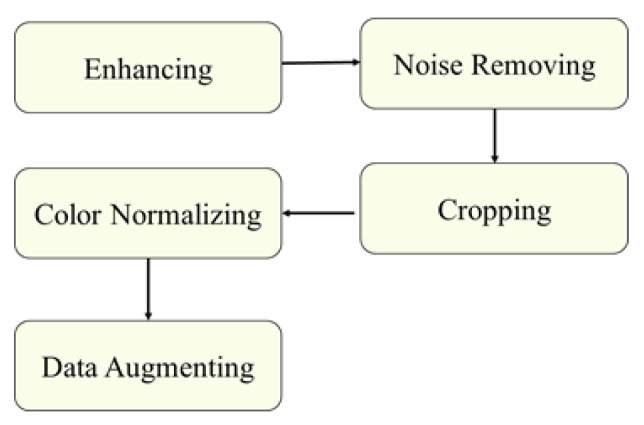
The retina images preprocessing methods.

**Figure 7:**
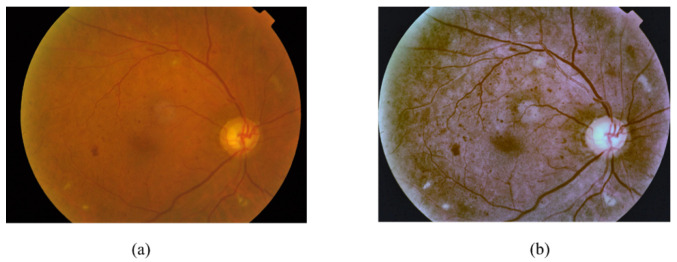
Sample images of the (**a**) original image and (**b**) the preprocessing image.

Image preprocessing is important for improving the quality of retinal images, since images with low quality can reduce the network’s performance [25] and it is necessary to ensure that all the images are consistent and that the features of the images are enhanced [52,53]. The applied preprocessing methods, shown in Figure 6, are as follows. The result of the preprocessing step is shown in Figure 7.

**Figure 8:**
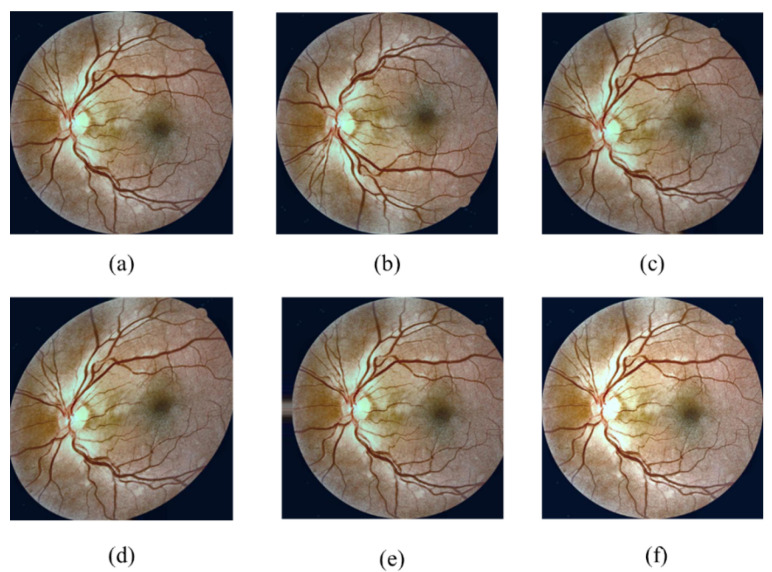
Sample of an image augmentation: (**a**) original image, (**b**) flipped image, (**c**) rotated image, (**d**) sheared image, (**e**) translated image and (**f**) brightened image.

- Image Enhancement: Two methods were used to enhance the images, the enhance luminosity method [54] and Contrast Limited Adaptive Histogram Equalization (CLAHE). CLAHE [55] is successful in enhancing the contrast of the fundus images [56] and improving the low contrast of medical images [57]. CLAHE is applied to the L channel of the retina images that have a higher contrast [44], with tile size 8 × 8 and Clip Limit 5.0.
- Image Noise Removal: The CLAHE method can cause some noise in the images [54] and, to remove this noise, we applied the Gaussian filter, as represented in Equation (1).
  (1) $$G_{0}\left(x,y\right)=Ae^{\frac{-{\left(x-\mu_{x}\right)}^{2}}{2\sigma_{x}^{2}}+\frac{-{\left(y-\mu_{y}\right)}^{2}}{2\sigma_{y}^{2}}}$$
  where μ is the mean, *A* is the amplitude and σ is the standard deviation of each of the variables *x* and *y*.
- Image Cropping: The images were cropped to eliminate the unnecessary black pixels around the retina. Thus, the bounding box lesion positions in the annotation files were changed. To fix that, we automated changing the bounding box position of each image based on the number of removed pixels around the retina.
- Colour Normalisation: The retina images were captured from patients of different age, and various ethnicity [58], at different levels of lighting in the fundus image. These conditions have an effect on the value of pixel intensity of each image and create unnecessary variation in the image [58]. To overcome this, the retina images were normalised by normalising each channel of RGB images. For the normalization, we subtract the mean, and after that, divide the variance of the images [25], as shown in Equation (2).
  (2) $$z=\frac{\left(x-u\right)}{s}$$
  where *x* is training RGB retina images, *u* is the mean of the RGB retina training images and *s* is the standard deviation of the training RGB retina images.
- Online data augmentation was adopted to enlarge the training dataset and to improve the generalisation and performance of the CNN. The images were augmented by performing rotation, flipping, shearing, and translation, as well as randomly darkening and brightening them, as shown in Figure 8. The augmentation parameters are presented in Table 5. Finally, the images were resized into a fixed size that varied according to the CNN used.
- Extract Lesions Patches: Some preprocessing methods were applied for Lesion localization Method to extract the lesion patches from each image for the CNN training. First, we cropped the annotated bounding box of each lesion and then padded it by zero if its size was less than (65 × 65); otherwise, we resized the patch to (65 × 65) to standardise the size of the patches.

In addition to the above preprocessing methods, we noticed that some of the image annotation files contained duplicate lesions. Thus, we automated the removal of the duplicate lesions in the annotation file as in Algorithm 1. Moreover, the bounding box of each lesion was enlarged by 10 pixels around each lesion to make the lesions clearer for learning. The chosen number was suitable for the resolution used.
**Algorithm** **1**: Automate detecting and removing duplicate lesions.
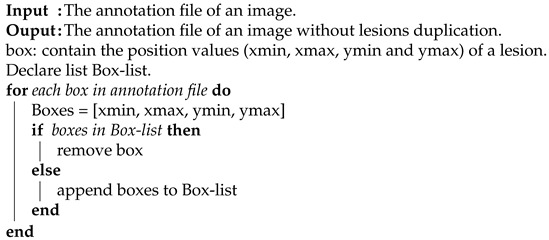


### 3.3. Image Based Method

This method takes the whole image as input to the CNN. The CNN architecture involves four main layers: convolution layers (CONV), pooling layers, fully connected layers (FC) and classification layer. The CONV layer role is to extract the features of the images by convolving different filters, while the pooling layer reduce the dimensions of the feature maps [59]. The FC layers are a compact feature to describe the whole input image. The Batch Normalisation layer role is to normalise the inputs of a layer during training to increase the training speed and regularise the CNN. We proposed two simple custom CNN models with different image sizes to classify the DR images. Moreover, EfficientNetB0 [12] was fine-tuned to classify the DR images.

#### 3.3.1. Designed CNN Model

We started designing the proposed CNN as similar CNN from related works like [26]. Then, we increased the input layer size to consider the MA lesion and the number of CNN layers were increased gradually to improve the CNN performance. We adjusted the hyperparameter as in Section 4. After many attempts with many CNNs architectures as described in Section 4.3, we improved the DR classification using the proposed CNN.

**Figure 9:**
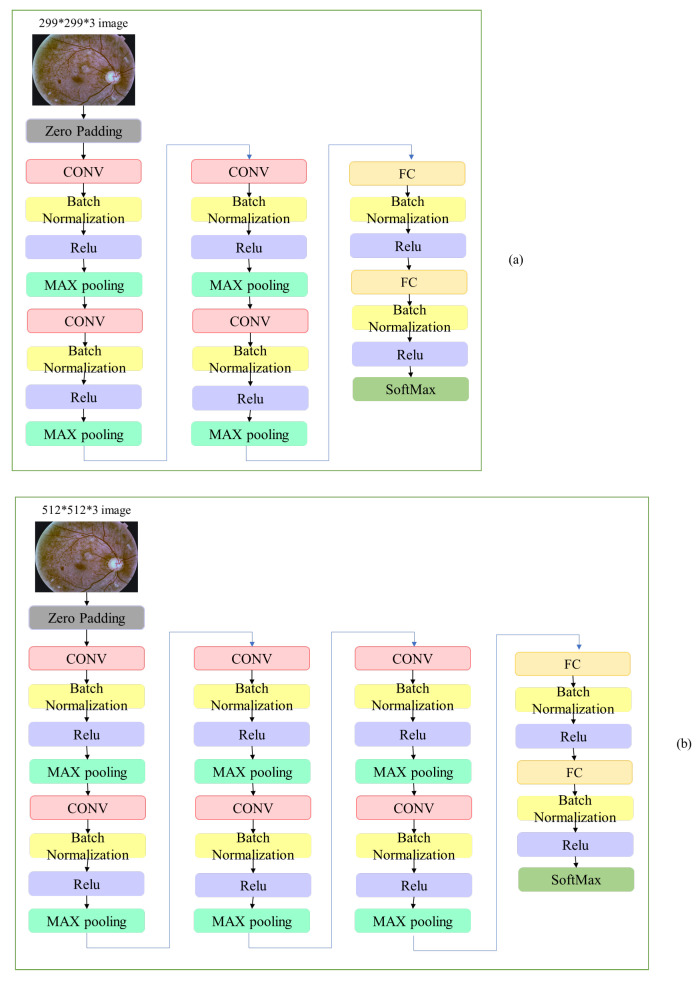
The proposed custom CNN architectures: (**a**) CNN299 and (**b**) CNN512.

The first proposed CNN (CNN299) contains one Zero Padding layer with a value of 2, four CONV layers, four Max Pooling layers, six Batch Normalization layers, two FC layers and one SoftMax layer for classification. The second proposed CNN (CNN512) contains one Zero Padding layer with a value of 2, six CONV layers, each followed by Max Pooling layers, eight Batch Normalization layers, two FC layers and one SoftMax layer for classification. The used input size of the CNNs was chosen to be suitable to the available computation power and it was not too small in order to avoid losing small lesions. The input image size was 299 × 299 × 3 for CNN299 and 512 × 512 × 3 for CNN512. The number of parameters of the CNN299 was 28,412,981 and for the CNN512 was 8,211,613. The CNN299 and CNN512 architectures are shown in Figure 9 and Table 6 and Table 7.

#### 3.3.2. Using Transfer Learning

**Figure 10:**
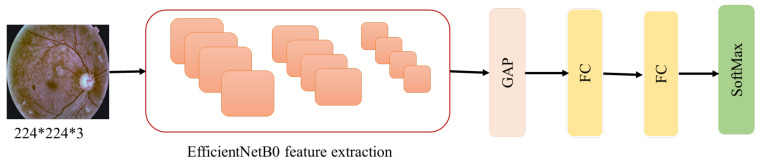
Transfer learning EfficientNetB0.

Transfer learning is a well-known machine learning technique in which a pre-trained neural network is used to solve a problem similar to what the network was initially designed and trained to solve. Transfer learning is a commonly used technique with deep learning as it can overcome many problems associated with deep neural networks. Using transfer learning can reduce the training time and tuning efforts for many hyperparameters [60]. It transfers the knowledge from a pretrained network that was trained on large training data to a target network in which limited training data are available [11]. There are two deep transfer learning strategies: feature extraction of pretrained models and fine-tuning the pretrained models [10]. EfficientNet is a pretrained network [12]. It is a recently proposed model and has achieved state-of-the-art results on the ImageNet dataset. EfficientNetB0 [12] was fine-tuned by initialising its weights with ImageNet weights and re-training all of its layers with the used retina datasets. The top layers of EfficientNetB0 were removed and replaced by new layers which are the Global Average Pooling (GAP) layer, two FC layers and SoftMax layer, as shown in Figure 10. At FC layers, we added Dropout with a rate of 0.5 in all used CNNs to overcome an overfitting problem.

### 3.4. Lesion Localization Method

The current work proposed two methods for the lesions localization: fine-tuning YOLOv3 [13] and cropping the images into small and fixed-size patches. YOLOv3 is a publicly available object detector model that predicts object bounding box (localise) and predict its class. YOLOv3 predicts objects from the whole image at three different outputs with three different scales in order to predict the object boxes. YOLOv3 contains 53 CONV layers formed in a network called Darknet-53 [13].

**Figure 11:**
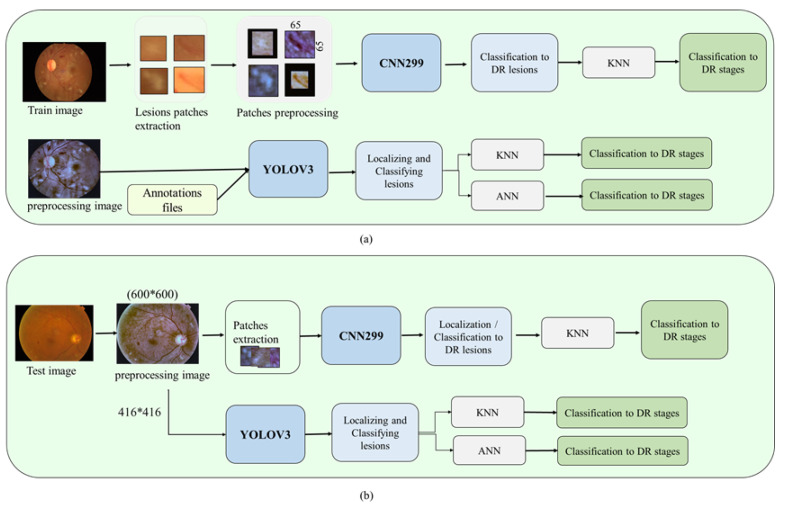
The proposed Lesion Localization method to detect DR stages and locate lesions for (**a**) train and (**b**) test images.

In the first method, all the YOLOv3 [13] layers were fine-tuned and re-trained using the preprocessed images of the DDR dataset, with an input size of 416 × 416 pixels to localise and classify all the DR lesions types. One dropout after layer 79 was added to improve the performance of YOLOv3. The second method to localise lesions is based on cropping the preprocessing images of the size 600 × 600 into 65 × 65 patches and then feeding them to CNN299 to classify them into different lesions types, as shown in Figure 11. The annotated lesions files were used to extract the lesion patches and then preprocess them. After that, these preprocessed patches were used to train the CNN299 from scratch to classify the various DR lesions of the DDR dataset. For detecting the non-lesions patches, we extracted patches from the non-DR images. Figure 11 illustrates the steps of the Lesion Localization method.

Moreover, the performance of classifying the images into DR stages based on the detected number of lesions types from Lesion localization Method was investigated by training three machine learning methods. Three different classifiers were tested to classify the DR stage according to the existence of various DR lesions. These classifiers were the k-nearest neighbors (KNN) [61], artificial neural networks (ANN) and the support vector machine (SVM). The ANN used contains three FC layers, with each FC followed by Batch Normalization layers. The last layer was the SoftMax layer for classification. The classification performance of localization method was compared with the Image Based Method. Finally, the robust classification method was fused with a strong localization method.

## 4. Experiments and Results

### 4.1. Configuration

The proposed system was implemented using the Python language and Keras framework [62] built on top of TensorFlow. All experiments were performed on two GPU resources: NVIDIA Tesla K20 GPU with 5 GB memory and NVIDIA GeForce 930 mx with 2 GB memory. The datasets were split into 80% for training and 20% for testing.

Deep learning network hyperparameters are variables that pre-select by a human designer or tuned via optimizing hyperparameters methods [63]. These methods involve random search [64], grid search [65], and gradient-based optimization [66]. We utilized manual hyperparameters tuning to speed up the process of tuning hyperparameters. The hyperparameter configuration of the used CNN models and YOLOv3 are shown in Table 8 and Table 9, respectively.

### 4.2. Performance Metrics

The metrics used to evaluate the performance of CNNs are accuracy (ACC), specificity (SP), sensitivity (SEN), Receiver Operating Characteristic (ROC) curve, Area Under the ROC Curve (AUC), positive predictive value (PPV) (also called Precision), Negative predictive value (NPV) and Disc similarity coefficient (DSC). ACC is the percentage of accurately classified images. SP is the percentage of images accurately classified as normal images, while SEN is the percentage of images accurately classified as DR images. The ratio between SEN and SP is graphically illustrated in the ROC curve and the value computed by ROC AUC. PPV is the percentage of DR images accurately classified as DR images while NPV is the percentage of normal images accurately classified as normal. The metrics used to evaluate the performance of YOLOv3 is Average precision (AP). The mean AP (mAP) is the average of the AP for each class. Each measurement is illustrated as follows.

(3) $$SP=\frac{TN}{\left(TN+FP\right)}$$

(4) $$SEN=\frac{TP}{\left(TP+FN\right)}$$

(5) $$ACC=\frac{\left(TN+TP\right)}{\left(TN+TP+FN+FP\right)}$$

(6) $$PPV=\frac{TP}{\left(TP+FP\right)}$$

(7) $$NPV=\frac{TN}{\left(TN+FN\right)}$$

(8) $$AP=\sum_{n}\left(R_{n}-R_{n-1}\right)P_{n}$$

where false positive (FP) refers to the non-DR images that are classified as DR, while false negative (FN) means the DR images that are classified as non-DR. True positive (TP) refers to the DR images that are classified as DR and true negative (TN) is the non-DR images that are classified as non-DR. $R_{n}$ and $P_{n}$ are the recall and the precision at the *n* threshold.

### 4.3. Image Based Method Results

Regarding the Image-Based Method, three CNN architectures were built for detecting the five DR stages: two custom CNNs, with different input sizes that were trained from scratch, and one fine-tuned EfficientNetB0. The CNNs were trained and tested on the DDR and the Kaggle APTOS 2019 datasets independently.

**Figure 12:**
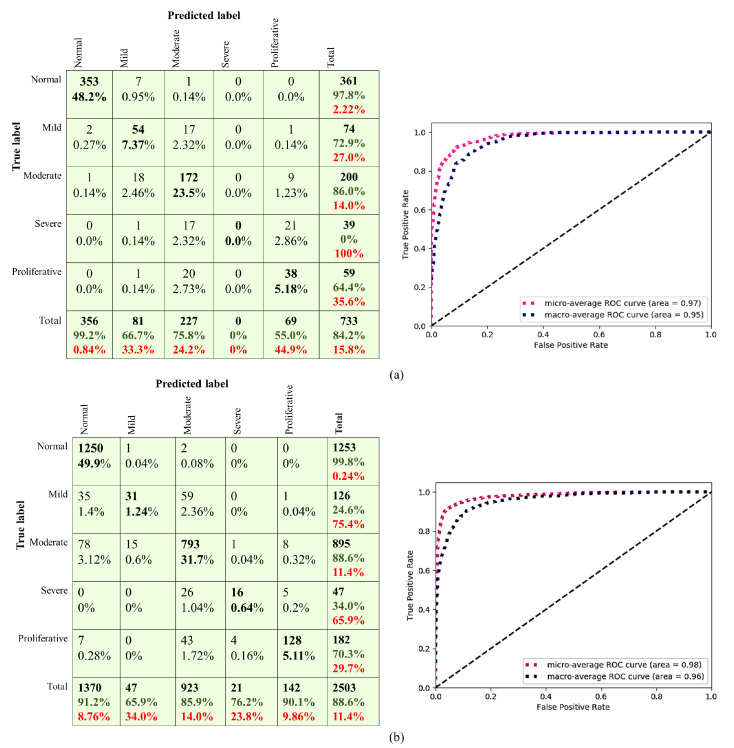
The ROC curves of the (**a**) APTOS 2019 and (**b**) the DDR datasets on CNN512.

In the experiments, the stochastic gradient descent (SGD) algorithm with the Nesterov Momentum was adopted. Moreover, Cyclical Learning Rates [67] with Learning rate range [$1\times 10^{-4}$, $1\times 10^{-1}$] and [$1\times 10^{-4}$, $1\times 10^{-2}$] were used for the custom CNNs and EfficientNetB0, respectively. The dropout at FC layers of the CNNs was implemented to reduce the overfitting and improve the CNNs’ performance. The distribution of all datasets’ classes was imbalanced and, to fix that, the class weight parameter was set to “auto”. The experiments showed that the CNN512 with the input size of 512 had a better performance than the other CNNs in both datasets. From Table 10 and Table 11, we found that the CNN512 with dropout achieved the highest ACC of 0.841 and 0.886 in the APTOS 2019 and the DDR datasets, respectively. The experiments also showed that the enhanced images luminosity method did not improve the classification accuracy when applied to the APTOS 2019 dataset with the CNN299 model, as shown in Table 11. Table 12 and Table 13 show the classification results of each DR stage from the APTOS 2019 and the DDR datasets, respectively. The ROC curves and confusion matrixes of the best proposed model results are shown in Figure 12.

### 4.4. Lesion Localization Method Results

YOLOv3 is trained on the DDR dataset to locate all DR lesions types and draws a bounding box around each lesion. YOLOv3 is trained using 608 images and tested using 149 images with 9 anchors. In the experiments, all YOLOv3 layers were retrained on the DDR dataset with a SGD optimizer, 0.9 momentum and fixed $1\times 10^{-3}$ learning rate. It was observed through the experiments that YOLOv3 with the learning rate $1\times 10^{-3}$ and one dropout after layer 79 had a better performance on the valid DDR dataset. YOLOv3 achieved the highest mAP of 0.216 at localising the DR lesions of the valid set when one dropout and the Adam optimizer were used, as shown in Table 14.

On the other hand, the KNN method obtained the best results for classifying the DR lesions into various DR stages, as in Table 15. The detected lesions by YOLOv3 and CNN299 were fed to the KNN or ANN to classify them into the different DR stages. When YOLOv3 and CNN299 did not detect any lesions, the image was classified as no DR stage. From Table 16, we found that the detected lesions from YOLOv3 with SGD and then classified by the KNN achieved the highest ACC of 0.712 in the valid set of the DDR dataset.

### 4.5. Comparison against State-of-the-Art Methods

Compared to the state of-the-art methods on the DDR and the APTOS 2019 datasets, our CNN512 achieved high results. Our CNN512 on the DDR dataset achieved a 0.886 ACC, while in the works of [32,37] achieved 0.828 and 0.856 ACC, respectively. In the APTOS 2019 dataset, our CNN512 achieved a 0.841 ACC, which is better than the works of [31,33,34]. The results of the CNNs in both datasets are shown in Table 10 and Table 11, respectively.

When compared to the results achieved by YOLOv3 on the DDR dataset with the state-of-the-art methods, YOLOv3 obtained better results. Table 17 shows that YOLOv3 achieved a better mAP on a valid set than the work of [37] that used Faster RCNN.

### 4.6. Models Fusion

**Figure 13:**
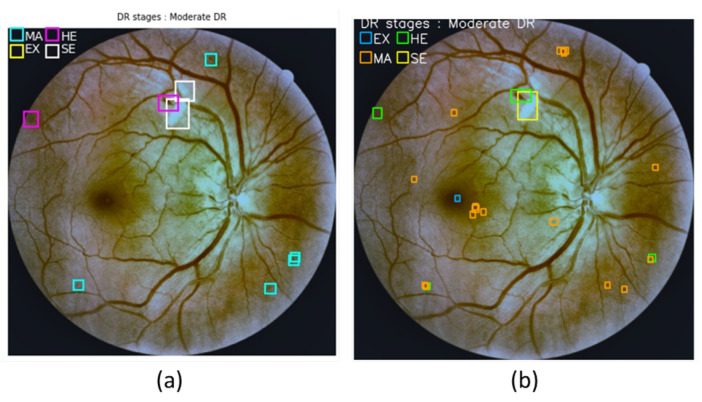
Sample of the DDR images visualization for: (**a**) the ground truth images annotation, (**b**) predicted images by fused model.

**Figure 14:**
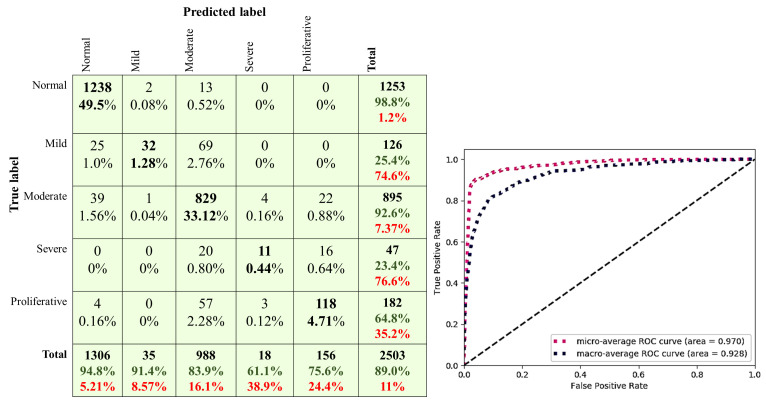
The confusion matrixes and ROC curves of the DDR dataset on fused models.

From the experiments, we found that the proposed CNN512 achieved the best DR stages classification results on the DDR dataset unlike the classification based on the detected DR lesions. Also, YOLOv3 classified and localised lesions on the retina with the best results. Thus, for classifying the retina images to the DR stages and localising DR lesions at the same time with the best results, CNN512 and YOLOv3 were fused. The classification predictions from the CNN512 model and YOLOv3 model with ANN were combined using average voting to fuse models. Average voting takes the average probabilities predicted from the two models as the final prediction result. When compared the results achieved by fused models on the DDR dataset with the state-of-the-art methods, the fused models obtained a 0.890 ACC exceeds the state-of-the-art results as shown in Table 10. Sample images visualization of lesions localising and stages classifying for the ground truth images and predicted images by the fused models are shown in Figure 13. The ROC curves and confusion matrixes of the fused models are shown in Figure 14. The average inference time for the fused models is 6.36 s using NVIDIA Tesla K20 GPU.

## 5. Discussion

Diabetic retinopathy (DR) is one of the most severe diabetes complications, causing non-reversible damage to retina blood vessels. Regular scanning using high-efficiency computer-based systems to diagnose cases early will help diabetes patients to stop or delay the deterioration of sight. This study proposed a DR screening system using the deep learning technique. The proposed screening system provides classification and DR lesions localization for DR images to help ophthalmologists diagnose the patients’ DR stage. The experimental results demonstrated that our custom CNN512 model achieved state-of-the-art classification results on the used two datasets.

**Figure 15:**
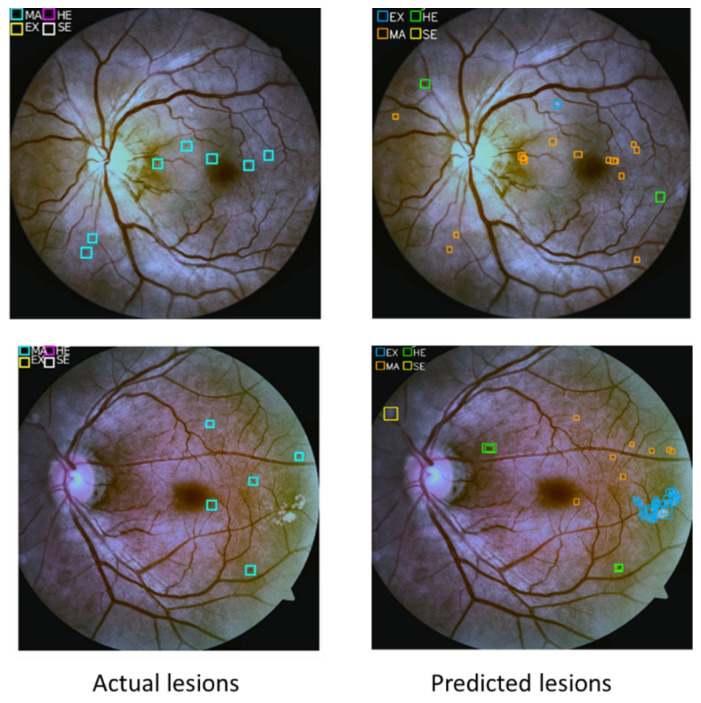
Samples of the miss labeled lesions from the DDR dataset compared to the predicted lesions by YOLOv3.

Furthermore, the fine-tuned YOLOv3 model obtained state-of-the-art localization results on the DDR dataset. CNN512 model and the fine-tuned YOLOv3 model were integrated to classify the DR images into stages and localize all lesion types. As we notice from the results, all of the models are slightly high with the DDR dataset rather than the APTOS dataset, which might result from the larger DDR training set. If a close look is taken on Table 12 and Table 13, it will be noticed that the sensitivity for mild and severe DR is lower than other stages; this resulted from the imbalance of the used datasets. For example, the mild class on DDR is less than 5% of the total dataset size; also, the severe stage image size is less than 2% of the DDR dataset. This limits the system performance for both mild and severe classes’ diagnoses, and it is reflected on PPV value even when we used the data augmentation technique to increase the data size. We inferred that, as the input image’s size increased, the model’s accuracy increased but this is limited with the available computing power. Some of the misclassified lesions in the images were examined and we found that spots detected on the retina by YOLOv3 were not in the ground truth lesions. The missed labeling of used images affected the results that the model obtained. Figure 15 shows samples of the incorrectly labeled lesions from the DDR dataset.

Recently, a new trend has appeared in DR which is developing a system that attempts to predict the development and change in DR over time as in [68,69]. In [68], they predicted future DR image using vessel and lesion information, achieving a 0.74 F1-score. In contrast, in [69], they evaluated the changes in DR using optimization algorithm and Support Vector Machines, obtaining a 95.23% ACC.

In the future, we could improve the localization of the lesions by creating a custom object detection model and by improving the performance classification of the CNN512 by adding more layers. Testing and tuning the system on more balanced datasets might improve its performance. In addition, we aim to adopt YOLOv4 and YOLOv5 to detect all DR lesions to obtain their benefits, such as ACC and speed. The current work opens the pathway to building a complete automatic follow-up system for DR. DR is a lifelong disease with a prolonged potential phase, so patients follow-up regularly will prevent patients’ blindness and delay sight deterioration. Table 18 Comparing the performance of the proposed models in term of accuracy.

## 6. Conclusions

The prevalence of diabetes is increasing worldwide, and the complication of DR is also increasing. This disorder is threatening diabetes patients’ vision if DR is detected in the last stages. Therefore, the detection and treatment of DR in its early stages is essential to decrease the risk of blindness. The manual diagnosis process of DR with the increasing suffering from DR became not sufficiently effective. Therefore, automating DR’s diagnosis using computer-aided screening systems (CASS) saves effort, time, and cost.

Additionally, the most critical point for using CASS is to avoid the negative impact of losing eyesight. Recently, the deep learning (DL) method has achieved superior performance in classification and segmentation. The current work provides an effective complete automated screening system to help in DR diagnosis. The quality and balance of the datasets used to build a DR screening system are very critical. In the future, we aim to combine multiple datasets to achieve the balance of the dataset.

## Figures and Tables

**Table 1:** The DR stages depending on lesions classification [4].

DR Severity Level	Lesions
No DR	No lesions.
Mild DR	MA only.
Moderate DR	More than just MA but less than severe DR.
Severe DR	Any of the following: more than 20 intraretinal HM in each of 4 quadrants; definite venous beading in 2+ quadrants; Prominent intraretinal microvascular abnormalities in 1+ quadrant and no signs of proliferative DR.
Proliferative DR	One or more of the following: neovascularization, pre-retinal HM.

**Table 2:** Comparison between the related works that used DL to classify DR Images.

Ref.	Number of Classes	Detect Lesion	Dataset	Performance Measure			
				AUC	ACC	SEN	SP
[15]	2	No	Messidor-2, DR2	98.2% 98%	-	-	-
[22]	2	No	private dataset, STARE	0.9823 0.951	94.23% 90.84%	90.94%	95.74%
[18]	2	No	private dataset	0.946	88.21%	85.57%	90.85%
[23]	2	No	private dataset	-	98.7%	0.996	98.2%
[24]	5	No	Kaggle	-	63.23%	-	-
[25]	5	No	Kaggle	0.978	95.6%	86.4%	97.4%
[26]	4	No	Messidor	-	98.15%	98.94%	97.87%
[27]	4	No	private dataset	-	96.5%	98.1%	98.9%
[28]	5	No	IDRiD	-	90.07%	-	-
[29]	4	No	Messidor	-	96.35	92.35	97.45
[30]	5	No	IDRiD	-	65.1%	-	-
[31]	5	No	APTOS 2019	-	0.77	-	-
[32]	5	No	Messidor, DDR, Kaggle	-	0.8408 0.8569 0.8668	-	-
[33]	5	No	APTOS 2019	-	83.09	88.24	87
[34]	5	No	APTOS 2019	-	82.54	83	-
[35]	3	No	private dataset, EYEPACS	0.955, 0.984, 0.955	-	-	-
[36]	2	Red lesion only	Messidor	0.912	-	0.94	-
[37]	5	Yes	DDR	-	0.8284	-	-
[38]	5	Red lesion only	private dataset, Messidor	- 0.972	92.95 -	99.39% 92.59%	99.93% 96.20%

**Table 3:** The DR datasets details.

	DDR	DDR Lesions Annotated	APTOS 2019 Kaggle
Training	10,019 images	606 images	2929 images
Testing	2503 images	149 images	733 images
No DR	6266 images	-	1805 images
Mild	630 images	99 images	370 images
Moderate	4477 images	548 images	999 images
Severe	236 images	34 images	193 images
Proliferative	913 images	74 images	295 images
Image Size	Different image size	Different image size	Different image size
Total	12,522 images	755 images	3662 images

**Table 4:** The annotated lesions distribution in the DDR dataset.

	MA Number	HM Number	Hard EX Number	Soft EX Number	Total
Training	7824	11,196	21,739	944	41,703
Testing	2556	1342	1920	349	6167
Total	10,380	12,538	23,659	1293	47,870

**Table 5:** Data augmentation parameters.

Transformation Type	Description
Rotation	Rotate the image randomly between ($-35^{\circ }$, 35${}^{\circ }$).
Flipping	Horizontal and vertical flip for the images.
Shearing	Randomly Shear images with angle between $-15^{\circ }$ and 15${}^{\circ }$.
Translation	Randomly with shift between −10% and 10% of pixels.
Brightness range	Randomly darken the image and brighten. The values less than 1.0 the image darken whereas values larger than 1.0 brighten the image. The used values (0.25, 1.25).

**Table 6:** The proposed CNN299 layers detail.

Layer	Operator	Layer Details
Input Layer	Zero Padding layer	Padding (2,2)
Layer 1	2D CONV layer	Kernel number = 32, kernel size = 3
Layer 2	Batch Normalization layer	-
Layer 3	Relu layer	-
Layer 4	Max Pooling layer	Pooling size (2,2)
Layer 5	2D CONV layer	Kernel number = 64, kernel size = 3
Layer 6	Batch Normalization layer	-
Layer 7	Relu layer	-
Layer 8	Max Pooling layer	Pooling size (2,2)
Layer 9	2D CONV layer	Kernel number = 96, kernel size = 3
Layer 10	Batch Normalization layer	-
Layer 11	Relu layer	-
Layer 12	Max Pooling layer	Pooling size (2,2)
Layer 13	2D CONV layer	Kernel number = 96, kernel size = 3
Layer 14	Batch Normalization layer	-
Layer 15	Relu layer	-
Layer 16	Max Pooling layer	Pooling size (2,2)
Layer 17	Flatten layer	-
Layer 18	FC layer	Neurons number = 1000
Layer 19	Batch Normalization layer	-
Layer 20	Relu layer	-
Layer 21	FC layer	Neurons number = 500
Layer 22	Batch Normalization layer	-
Layer 23	Relu layer	-
Layer 24	FC layer	With SoftMax activation

**Table 7:** The proposed CNN512 layers detail.

Layer	Operator	Layer Details
Input Layer	Zero Padding layer	Padding (2,2)
Layer 1	2D CONV layer	Kernel number = 32, kernel size = 3
Layer 2	Batch Normalization layer	-
Layer 3	Relu layer	-
Layer 4	Max Pooling layer	Pooling size (2,2)
Layer 5	2D CONV layer	Kernel number = 64, kernel size = 3
Layer 6	Batch Normalization layer	-
Layer 7	Relu layer	-
Layer 8	Max Pooling layer	Pooling size (2,2)
Layer 9	2D CONV layer	Kernel number = 96, kernel size = 3
Layer 10	Batch Normalization layer	-
Layer 11	Relu layer	-
Layer 12	Max Pooling layer	Pooling size (2,2)
Layer 13	2D CONV layer	Kernel number = 96, kernel size = 3
Layer 14	Batch Normalization layer	-
Layer 15	Relu layer	-
Layer 16	Max Pooling layer	Pooling size (2,2)
Layer 17	2D CONV layer	Kernel number = 128, kernel size = 3
Layer 18	Batch Normalization layer	-
Layer 19	Relu layer	-
Layer 20	Max Pooling layer	Pooling size (2,2)
Layer 21	2D CONV layer	Kernel number = 200, kernel size = 3
Layer 22	Batch Normalization layer	-
Layer 23	Relu layer	-
Layer 24	Max Pooling layer	Pooling size (2,2)
Layer 25	Flatten layer	-
Layer 26	FC layer	Neurons number = 1000
Layer 27	Batch Normalization layer	-
Layer 28	Relu layer	-
Layer 29	FC layer	Neurons number = 500
Layer 30	Batch Normalization layer	-
Layer 31	Relu layer	-
Layer 32	FC layer	With SoftMax activation

**Table 8:** The hyperparameter configuration of CNNs.

Configuration	Values
Optimizer	SGD
Momentum	0.9
Max Learning rate	$1\times 10^{-1}$ in custom CNNs $1\times 10^{-2}$ in EfficientNetB0
Base Learning rate	$1\times 10^{-4}$
Mode	triangular
Class weight	auto
Dropout	0.5
Augmentation	20 times

**Table 9:** The YOLOv3 hyperparameter configuration.

Configuration	Values
Optimizer	SGD and Adam
Momentum	0.9
Learning rate	$1\times 10^{-3}$
Anchors number	9
Augmentation	5 times
Input size	(416,416,3)
CNN model	Darknet53
Object threshold	0.45
NMS threshold	0.45
Dropout	0.5

**Table 10:** Comparison between the proposed models and the state-of-the-art models on the DDR dataset.

Model	Image Size	ACC	SEN	SP	AUC
Tao Li et al. [37]	224	0.828	-	-	-
Along He et al. [32]	512	0.856	-	-	-
CNN299	299	0.800	-	-	-
CNN299 + dropout	299	0.833	-	-	-
CNN512	512	0.858	0.858	0.963	0.975
CNN512 + dropout	512	0.886	0.886	0.971	0.979
EfficientNetB0	224	0.823	-	-	-
EfficientNetB0 + dropout	224	0.822	-	-	-
Models fusion	512	0.890	0.890	0.973	0.970

**Table 11:** Comparison between the proposed models and the state of-art models on the APTOS 2019 dataset.

Model	Image Size	ACC	SEN	SP	AUC
Omar Dekhil et al. [31]	224	0.77	-	-	-
kassani et al. [33]	600	83.09	88.2	87.0	-
Bodapati et al. [34]	-	82.54	83	-	-
CNN299	299	0.821	-	-	-
CNN299 + dropout	299	0.832	-	-	-
CNN299 + dropout + enhance luminosity	299	0.832	-	-	-
CNN512	512	0.834	0.834	0.957	0.97
CNN512 + dropout	512	0.841	0.841	0.960	0.973
EfficientNetB0	224	0.823	-	-	-
EfficientNetB0 + dropout	224	0.822	-	-	-

**Table 12:** The performance measures of the DR stages using CNN512 for the APTOS 2019 dataset.

Stage	SEN	SP	PPV	NPV
No DR	0.978	0.991	0.991	0.979
Mild DR	0.730	0.959	0.667	0.969
Moderate DR	0.860	0.897	0.758	0.945
Severe DR	0	100	0	0.947
Proliferative DR	0.644	0.954	0.550	0.968

**Table 13:** The performance measures of the DR stages using CNN512 for the DDR dataset.

Stage	SEN	SP	PPV	NPV
No DR	0.998	0.904	0.912	0.997
Mild DR	0.246	0.993	0.660	0.961
Moderate DR	0.886	0.919	0.859	0.935
Severe DR	0.340	0.998	0.762	0.988
Proliferative DR	0.703	0.993	0.901	0.977

**Table 14:** Results of YOLOv3 on the DDR Dataset.

Model	mAP
YOLOv3 + SGD	0.110
YOLOv3 + SGD+ dropout	0.171
YOLOv3 + Adam optimizer + dropout	0.216

**Table 15:** The DR stages classification training results using machine learning.

Model	ACC
KNN	0.985
ANN	0.893
SVM	0.872

**Table 16:** The results of DR stages classification using Lesion localization Method on the DDR dataset.

Model	Valid Images Number	ACC	SEN	SP	AUC
CNN299 + KNN	250 images	0.62	0.62	0.90	0.762
YOLOv3 + SGD+ dropout + KNN	250 images	0.712	0.712	0.928	0.820
YOLOv3 + Adam+ dropout + KNN	250 images	0.552	0.552	0.888	0.720
YOLOv3 + SGD+ dropout + KNN	2503 images	0.528	0.528	0.882	0.705
YOLOv3 + ADAM+ dropout +ANN	2503 images	0.481	0.481	0.870	0.789

**Table 17:** Comparison between the YOLOv3 model and the state of-art models on the DDR dataset.

Model	mAP
Tao li et al. [37]	0.092
YOLOv3 + Adam optimizer + dropout	0.216

**Table 18:** The performance comparison among all of the models.

Model	EfficientNetB0 + Dropout		CNN299 + Dropout		CNN512 + Dropout		Model Fusion
Dataset	APTOS	DDR	APTOS	DDR	APTOS	DDR	DDR
ACC	0.822	0.822	0.832	0.833	0.841	**0.886**	**0.890**

## Data Availability

Publicly available datasets were used in this study. DDR dataset at https://doi.org/10.1016/j.ins.2019.06.011 accessed 28 August 2019 and APTOS 2019 Blindness Detection at https://www.kaggle.com/c/aptos2019-blindness-detection/overview/evaluation accessed 1 January 2020.

## References

[1] American Academy of Ophthalmology-What Is Diabetic Retinopathy. https://www.aao.org/eye-health/diseases/what-is-diabetic-retinopathy.

[2] Bourne R.R., Stevens G.A., White R.A., Smith J.L., Flaxman S.R., Price H., Jonas J.B., Keeffe J., Leasher J., Naidoo K. (2013). Causes of vision loss worldwide, 1990-2010: A systematic analysis. Lancet Glob. Health.

[3] Taylor R., Batey D. (2012). Handbook of Retinal Screening in Diabetes: Diagnosis and Management.

[4] Wilkinson C.P., Ferris F.L., Klein R.E., Lee P.P., Agardh C.D., Davis M., Dills D., Kampik A., Pararajasegaram R., Verdaguer J.T. (2003). Proposed international clinical diabetic retinopathy and diabetic macular edema disease severity scales. Am. Acad. Ophthalmol..

[5] Deng L., Yu D. (2014). Deep learning: Methods and applications. Found. Trends Signal Process..

[6] Vega R., Sanchez-Ante G., Falcon-Morales L.E., Sossa H., Guevara E. (2015). Retinal vessel extraction using lattice neural networks with dendritic processing. Comput. Biol. Med..

[7] Al Zaid E., Shalash W.M., Abulkhair M.F. Retinal blood vessels segmentation using Gabor filters. Proceedings of the 2018 1st International Conference on Computer Applications & Information Security (ICCAIS).

[8] Sikder N., Masud M., Bairagi A.K., Arif A.S.M., Nahid A.A., Alhumyani H.A. (2021). Severity Classification of Diabetic Retinopathy Using an Ensemble Learning Algorithm through Analyzing Retinal Images. Symmetry.

[9] Bakator M., Radosav D. (2018). Deep learning and medical diagnosis: A review of literature. Multimodal Technol. Interact..

[10] Litjens G., Kooi T., Bejnordi B.E., Setio A.A.A., Ciompi F., Ghafoorian M., van der Laak J.A., van Ginneken B., Sánchez C.I. (2017). A survey on deep learning in medical image analysis. Med. Image Anal..

[11] Lu L., Zheng Y., Carneiro G., Yang L. (2017). Deep Learning and Convolutional Neural Networks for Medical Image Computing.

[12] Tan M., Le Q.V. EfficientNet: Rethinking model scaling for convolutional neural networks. Proceedings of the 36th International Conference on Machine Learning, ICML 2019.

[13] Redmon J., Farhadi A. (2018). YOLOv3: An Incremental Improvement. arXiv.

[14] Pal P., Kundu S., Dhara A.K. (2020). Detection of red lesions in retinal fundus images using YOLO V3. Curr. Indian Eye Res. J. Ophthalmic Res. Group.

[15] Pires R., Avila S., Wainer J., Valle E., Abramoff M.D., Rocha A. (2019). A data-driven approach to referable diabetic retinopathy detection. Artif. Intell. Med..

[16] Kaggle 2015 Dataset. https://kaggle.com/c/diabetic-retinopathy-detection.

[17] Decenciere E., Zhang X., Cazuguel G., Lay B., Cochener B., Trone C., Gain P., Ordonez R., Massin P., Erginay A. (2014). Feedback on a publicly distributed image database: The messidor database. Image Anal. Stereol..

[18] Jiang H., Yang K., Gao M., Zhang D., Ma H., Qian W. An Interpretable Ensemble Deep Learning Model for Diabetic Retinopathy Disease Classification. Proceedings of the 2019 41st Annual International Conference of the IEEE Engineering in Medicine and Biology Society (EMBC).

[19] Szegedy C., Ioffe S., Vanhoucke V., Alemi A.A. Inception-v4, inception-resnet and the impact of residual connections on learning. Proceedings of the Thirty-First AAAI Conference on Artificial Intelligence.

[20] Szegedy C., Vanhoucke V., Ioffe S., Shlens J., Wojna Z. Rethinking the Inception Architecture for Computer Vision. Proceedings of the IEEE Conference on Computer Vision and Pattern Recognition.

[21] He K., Zhang X., Ren S., Sun J. Deep Residual Learning for Image Recognition. Proceedings of the IEEE Conference on Computer Vision and Pattern Recognition.

[22] Liu Y.P., Li Z., Xu C., Li J., Liang R. (2019). Referable diabetic retinopathy identification from eye fundus images with weighted path for convolutional neural network. Artif. Intell. Med..

[23] Das S., Kharbanda K., Suchetha M., Raman R., Dhas E. (2021). Deep learning architecture based on segmented fundus image features for classification of diabetic retinopathy. Biomed. Signal Process. Control.

[24] Wang X., Lu Y., Wang Y., Chen W.B. Diabetic retinopathy stage classification using convolutional neural networks. Proceedings of the International Conference on Information Reuse and Integration for Data Science.

[25] Wan S., Liang Y., Zhang Y. (2018). Deep convolutional neural networks for diabetic retinopathy detection by image classification. Comput. Electr. Eng..

[26] Mobeen-Ur-Rehman, Khan S.H., Abbas Z., Danish Rizvi S.M. Classification of Diabetic Retinopathy Images Based on Customised CNN Architecture. Proceedings of the 2019 Amity International Conference on Artificial Intelligence, AICAI 2019.

[27] Zhang W., Zhong J., Yang S., Gao Z., Hu J., Chen Y., Yi Z. (2019). Automated identification and grading system of diabetic retinopathy using deep neural networks. Knowl. Based Syst..

[28] Harangi B., Toth J., Baran A., Hajdu A. Automatic screening of fundus images using a combination of convolutional neural network and hand-crafted features. Proceedings of the 2019 41st Annual International Conference of the IEEE Engineering in Medicine and Biology Society (EMBC).

[29] Shanthi T., Sabeenian R.S. (2019). Modified Alexnet architecture for classification of diabetic retinopathy images. Comput. Electr. Eng..

[30] Li X., Hu X., Yu L., Zhu L., Fu C.W., Heng P.A. (2020). CANet: Cross-disease Attention Network for Joint Diabetic Retinopathy and Diabetic Macular Edema Grading. IEEE Trans. Med Imaging.

[31] Dekhil O., Naglah A., Shaban M., Ghazal M., Taher F., Elbaz A. Deep Learning Based Method for Computer Aided Diagnosis of Diabetic Retinopathy. Proceedings of the IST 2019—IEEE International Conference on Imaging Systems and Techniques.

[32] He A., Li T., Li N., Wang K., Fu H. (2020). CABNet: Category Attention Block for Imbalanced Diabetic Retinopathy Grading. IEEE Trans. Med. Imaging.

[33] Kassani S.H., Kassani P.H., Khazaeinezhad R., Wesolowski M.J., Schneider K.A., Deters R. Diabetic retinopathy classification using a modified xception architecture. Proceedings of the 2019 IEEE International Symposium on Signal Processing and Information Technology (ISSPIT).

[34] Bodapati J.D., Shaik N.S., Naralasetti V. (2021). Composite deep neural network with gated-attention mechanism for diabetic retinopathy severity classification. J. Ambient. Intell. Humaniz. Comput..

[35] Hsieh Y.T., Chuang L.M., Jiang Y.D., Chang T.J., Yang C.M., Yang C.H., Chan L.W., Kao T.Y., Chen T.C., Lin H.C. (2021). Application of deep learning image assessment software VeriSee™ for diabetic retinopathy screening. J. Formos. Med Assoc..

[36] Zago G.T., Andreão R.V., Dorizzi B., Teatini Salles E.O. (2019). Diabetic retinopathy detection using red lesion localization and convolutional neural networks. Comput. Biol. Med..

[37] Li T., Gao Y., Wang K., Guo S., Liu H., Kang H. (2019). Diagnostic assessment of deep learning algorithms for diabetic retinopathy screening. Inf. Sci..

[38] Wang J., Luo J., Liu B., Feng R., Lu L., Zou H. (2020). Automated diabetic retinopathy grading and lesion detection based on the modified R-FCN object-detection algorithm. IET Comput. Vis..

[39] Krizhevsky A., Sutskever I., Hinton G.E. (2017). ImageNet Classification with Deep Convolutional Neural Networks. Commun. ACM.

[40] Zisserman K.S., Zisserman A. Very deep convolutional networks for large-scale image recognition. Proceedings of the International Conference on Learning Representations.

[41] Szegedy C., Liu W., Jia Y., Sermanet P., Reed S., Anguelov D., Erhan D., Vanhoucke V., Rabinovich A. Going Deeper with Convolutions. Proceedings of the IEEE Conference on Computer Vision and Pattern Recognition (CVPR).

[42] He K., Zhang X., Ren S., Sun J. (2015). Spatial Pyramid Pooling in Deep Convolutional Networks for Visual Recognition. IEEE Trans. Pattern Anal. Mach. Intell..

[43] Chollet F. Xception: Deep learning with depthwise separable convolutions. Proceedings of the IEEE Conference on Computer Vision and Pattern Recognition (CVPR).

[44] Huang G., Liu Z., van der Maaten L., Weinberger K.Q. Densely Connected Convolutional Networks. Proceedings of the IEEE Conference on Computer Vision and Pattern Recognition (CVPR).

[45] Porwal P., Pachade S., Kamble R., Kokare M., Deshmukh G., Sahasrabuddhe V., Meriaudeau F. (2018). Indian Diabetic Retinopathy Image Dataset (IDRiD): A Database for Diabetic Retinopathy Screening Research. Data.

[46] APTOS 2019 Blindness Detection. https://www.kaggle.com/c/aptos2019-blindness-detection/overview/evaluation.

[47] Alyoubi W.L., Shalash W.M., Abulkhair M.F. (2020). Diabetic Retinopathy Detection through Deep Learning Technique: A Review. Inform. Med. Unlocked.

[48] Hu J., Shen L., Sun G. Squeeze-and-excitation networks. Proceedings of the IEEE Conference on Computer Vision and Pattern Recognition.

[49] Ren S., He K., Girshick R., Sun J. (2017). Faster R-CNN: Towards real-time object detection with region proposal networks. IEEE Trans. Pattern Anal. Mach. Intell..

[50] Dai J., Li Y., He K., Sun J. (2016). R-FCN: Object detection via region-based fully convolutional networks. arXiv.

[51] Li T., Bo W., Hu C., Kang H., Liu H., Wang K., Fu H. (2021). Applications of Deep Learning in Fundus Images: A Review. Med. Image Anal..

[52] Esfahani M.T., Ghaderi M., Kafiyeh R. (2018). Classification of diabetic and normal fundus images using new deep learning method. Leonardo Electron. J. Pract. Technol..

[53] Dutta S., Manideep B.C., Basha S.M., Caytiles R.D., Iyengar N.C.S.N. (2018). Classification of Diabetic Retinopathy Images by Using Deep Learning Models. Int. J. Grid Distrib. Comput..

[54] Zhou M., Jin K., Wang S., Ye J., Qian D. (2018). Color Retinal Image Enhancement Based on Luminosity and Contrast Adjustment. IEEE Trans. Biomed. Eng..

[55] Pisano E.D., Zong S., Hemminger B.M., Deluca M., Johnston R.E., Muller K., Braeuning M.P., Pizer S.M. (1998). Contrast Limited Adaptive Histogram Equalization Image Processing to Improve the Detection of Simulated Spiculations in Dense Mammograms. J. Digit. Imaging.

[56] Zuiderveld K. (1994). Contrast Limited Adaptive Histogram Equalization. Graph. Gems IV.

[57] Sonali, Sahu S., Singh A.K., Ghrera S., Elhoseny M. (2019). An approach for de-noising and contrast enhancement of retinal fundus image using CLAHE. Optics Laser Technol..

[58] Pratt H., Coenen F., Broadbent D.M., Harding S.P., Zheng Y. (2016). Convolutional Neural Networks for Diabetic Retinopathy. Procedia Comput. Sci..

[59] Ketkar N. (2017). Deep Learning with Python.

[60] Shalash W.M. Driver Fatigue Detection with Single EEG Channel Using Transfer Learning. Proceedings of the 2019 IEEE International Conference on Imaging Systems and Techniques (IST).

[61] COVER T., HART P. (1967). Nearest Neighbor Pattern Classfication. IEEE Trans. Inf. Theory.

[62] Keras. https://keras.io/.

[63] Lee W.Y., Park S.M., Sim K.B. (2018). Optimal hyperparameter tuning of convolutional neural networks based on the parameter-setting-free harmony search algorithm. Optik.

[64] Bergstra J., Bengio Y. (2012). Random search for hyper-parameter optimization. J. Mach. Learn. Res..

[65] Huang Q., Mao J., Liu Y. An improved grid search algorithm of SVR parameters optimization. Proceedings of the 2012 IEEE 14th International Conference on Communication Technology.

[66] Maclaurin D., Duvenaud D., Adams R. Gradient-based hyperparameter optimization through reversible learning. Proceedings of the International Conference on Machine Learning, PMLR.

[67] Smith L.N. Cyclical learning rates for training neural networks. Proceedings of the IEEE Winter Conference on Applications of Computer Vision, WACV 2017.

[68] Ahn S., Pham Q., Shin J., Song S.J. (2021). Future Image Synthesis for Diabetic Retinopathy Based on the Lesion Occurrence Probability. Electronics.

[69] Anton N., Dragoi E.N., Tarcoveanu F., Ciuntu R.E., Lisa C., Curteanu S., Doroftei B., Ciuntu B.M., Chiseliţă D., Bogdănici C.M. (2021). Assessing Changes in Diabetic Retinopathy Caused by Diabetes Mellitus and Glaucoma Using Support Vector Machines in Combination with Differential Evolution Algorithm. Appl. Sci..

[70] Aziz Computer. http://hpc.kau.edu.sa.

